# Mass spectrometry analysis and transcriptome sequencing reveal glowing squid crystal proteins are in the same superfamily as firefly luciferase

**DOI:** 10.1038/srep27638

**Published:** 2016-06-09

**Authors:** Gregory Gimenez, Peter Metcalf, Neil G. Paterson, Miriam L. Sharpe

**Affiliations:** 1 Otago Genomics & Bioinformatics Facility, University of Otago, Dunedin, New Zealand; 2 School of Biological Sciences, University of Auckland, Auckland, New Zealand; 3 Diamond Light Source, Harwell Science and Innovation Campus, Didcot OX11 0DE, UK; 4 Department of Biochemistry, University of Otago, Dunedin, New Zealand

## Abstract

The Japanese firefly squid Hotaru-ika (*Watasenia scintillans*) produces intense blue light from photophores at the tips of two arms. These photophores are densely packed with protein microcrystals that catalyse the bioluminescent reaction using ATP and the substrate coelenterazine disulfate. The squid is the only organism known to produce light using protein crystals. We extracted microcrystals from arm tip photophores and identified the constituent proteins using mass spectrometry and transcriptome libraries prepared from arm tip tissue. The crystals contain three proteins, wsluc1–3, all members of the ANL superfamily of adenylating enzymes. They share 19 to 21% sequence identity with firefly luciferases, which produce light using ATP and the unrelated firefly luciferin substrate. We propose that wsluc1–3 form a complex that crystallises inside the squid photophores, and that in the crystal one or more of the proteins catalyses the production of light using coelenterazine disulfate and ATP. These results suggest that ANL superfamily enzymes have independently evolved in distant species to produce light using unrelated substrates.

Each spring, the bioluminescent firefly squid Hotaru-ika (*Watasenia scintillans*) provide a spectacular show of brilliant blue light as they come close to shore to spawn in Toyama Bay on the west coast of Japan. The 6 to 7 cm long squid (Fig. 1) emit light from three different types of photophores, the brightest of which are the three large organs found on the tips of each of the fourth pair of arms. These produce an intense blue light that is clearly visible in daylight if the squid are disturbed. Five smaller light organs are positioned in a row along the ventral margin of each eye, and hundreds of minute cutaneous photophores are scattered across the ventral integument of the mantle, funnel, head, and arms^1^,^2^,^3^. The smaller photophores emit weak light that is either blue or green (10% to 18% green, 82% to 90% blue^1^).

Biochemical analyses of the bioluminescent reaction have been challenging because the squid are only available for a few weeks each year, are difficult to maintain, and because light emission from dissected tissues is short-lived^4^. Nevertheless, research has established that coelenterazine disulfate is the *W. scintillans* luciferin substrate, and the reaction requires ATP, Mg^2+^ and molecular oxygen^3^,^4^,^5^,^6^,^7^,^8^,^9^. Difficulties in the isolation of active, soluble enzyme have led to researchers concluding that the luciferase was insoluble and membrane-bound^3^,^8^, or formed part of a cellular particle^4^.

The insoluble nature of the luciferase made the classical luciferin-lucifease experiment difficult; however, Tsuji clearly demonstrated that *W. scintillans* bioluminescence is produced by a luciferin – luciferase reaction^3^,^8^.

As early as 1927, the squid arm tip photophores were reported to contain numerous densely packed, tiny rod-shaped objects^10^. These were shown not to be bacteria (symbiotic bioluminescent bacteria are well known in other squid species) but possibly protein crystals, using microchemical techniques^11^ and electron microscopy^2^. In 2011, Hamanaka *et al*. confirmed that the rod-shaped objects are protein crystals, and demonstrated that they are the source of light production in the squid arm tip photophores^12^. This is the only report of naturally occurring protein crystals that are able to catalyse a bioluminescent reaction. They purified the ~5 × 2 μm crystals by centrifugation on sucrose gradients and analysed them by SDS-PAGE, revealing two bands. From this it was inferred that the crystals contain a major 63 kDa protein and a minor 81 kDa protein, in a mass ratio of about 8 to 1. Powder X-ray diffraction patterns demonstrated that the protein crystals are well ordered, diffracting to a resolution of up to about 15 Å.

In this study we have used a combination of high-throughput sequencing of protein-encoding mRNA transcripts from arm tip tissue and mass spectrometry of crystal extracts to identify three homologous proteins that comprise the luminescent arm tip microcrystals (wsluc1–3). These are all members of the ANL superfamily of adenylating enzymes (Acyl-CoA synthetases, Nonribosomal peptide-synthetase (NRPS) adenylation domains, firefly Luciferase). Sequencing of mantle tissue mRNA also revealed a close homolog of these proteins (wsluc4), which may be involved in cutaneous photophore bioluminescence. Our results reveal unexpected evolutionary convergence in the molecular mechanisms of bioluminescence and provide a basis for future investigations into how the microcrystals produce light.

## Results

### Sequencing, read cleaning and *de novo* assemblies

In order to obtain genome-wide protein sequence data for *W. scintillans*, for which only mitochondrial genome sequence data were previously available^13^, we sequenced the protein-encoding transcriptomes of two tissues that contain photophores: the tips of the fourth arms and the mantle. Total RNA was extracted from six samples: four separate arm tips, each including three large photophores, and two pieces of mantle containing small cutaneous photophores. After mRNA isolation and cDNA library construction, we sequenced the samples using an Illumina HiSeq-2000 sequencer. Sequencing generated 39.5 to 53.2 million pairs of 100 base length paired-end reads for each library (see Supplementary Table S1 for details). Adapter sequences were removed, low quality bases (Phred score <20) were trimmed from both ends of reads, and paired end reads less than 50 bases in length were discarded. Each library then contained 27.9 to 40.0 million high quality reads (70.8% to 74.6% of total raw reads).

**Figure 2:**
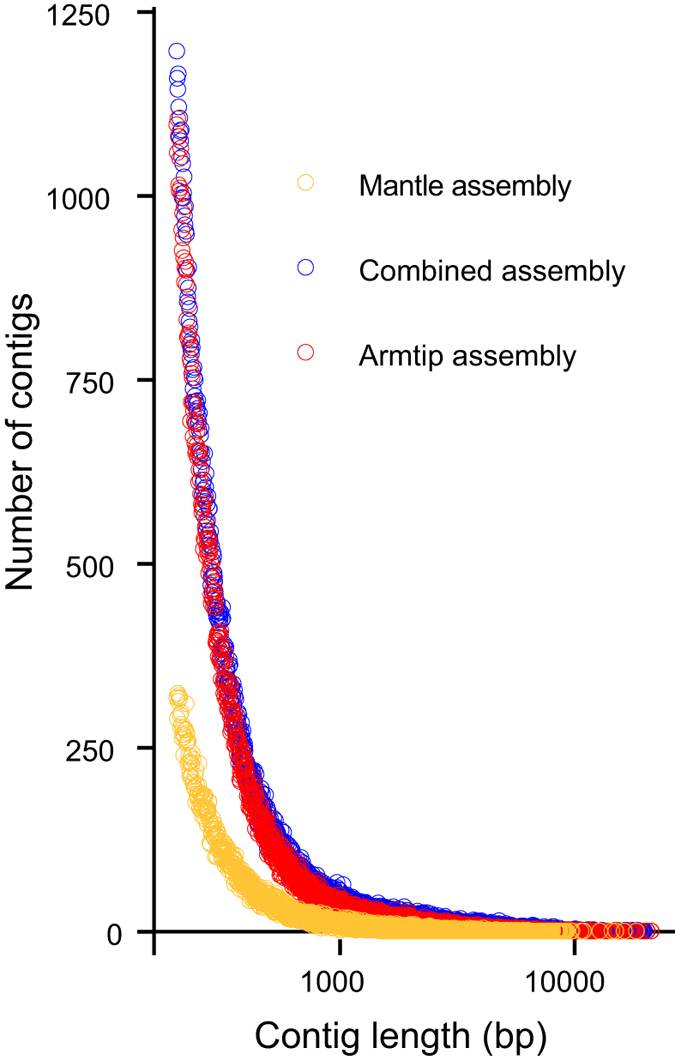
Distribution of contig lengths for arm tip, mantle and combined transcript assemblies.

Three *de novo* transcript assemblies were produced: one from the four arm tip libraries merged together, one from the two mantle libraries merged together, and a combined assembly from all six libraries merged together. Statistics for the assemblies are listed in Supplementary Table S2. The combined assembly had higher N50, median, mean and maximum values, and also contained more bases (239,722,196) and contigs (216,539) than the other two assemblies (148,602,829 bases and 178,970 contigs, and 36,531,652 bases and 53,406 contigs for the arm tip and mantle assemblies respectively). The contig length distribution was similar between the arm tip and combined assemblies and somewhat lower for the mantle assembly (Fig. 2) consistent with the lower RNA Integrity Number (RIN) values for the mantle samples.

### Identification of arm tip crystal proteins using SDS-PAGE and mass spectrometry

We observed that crystals dissolved rapidly during photophore dissection and subsequent purification, and tested a variety of storage solutions to stabilise them including sucrose, glycerol, hexylene glycol (MPD), ethylene glycol and polyethylene glycol (PEG) 400. We were able to stabilise the crystals in 40% sucrose in PBS for long enough to complete extraction. Crystals stored in this solution at 4 °C dissolved two to five days after extraction. The crystals also dissolved when flash cooled in liquid nitrogen and thawed on ice or at room temperature, using 40% sucrose in PBS as a cryoprotectant.

**Figure 3:**
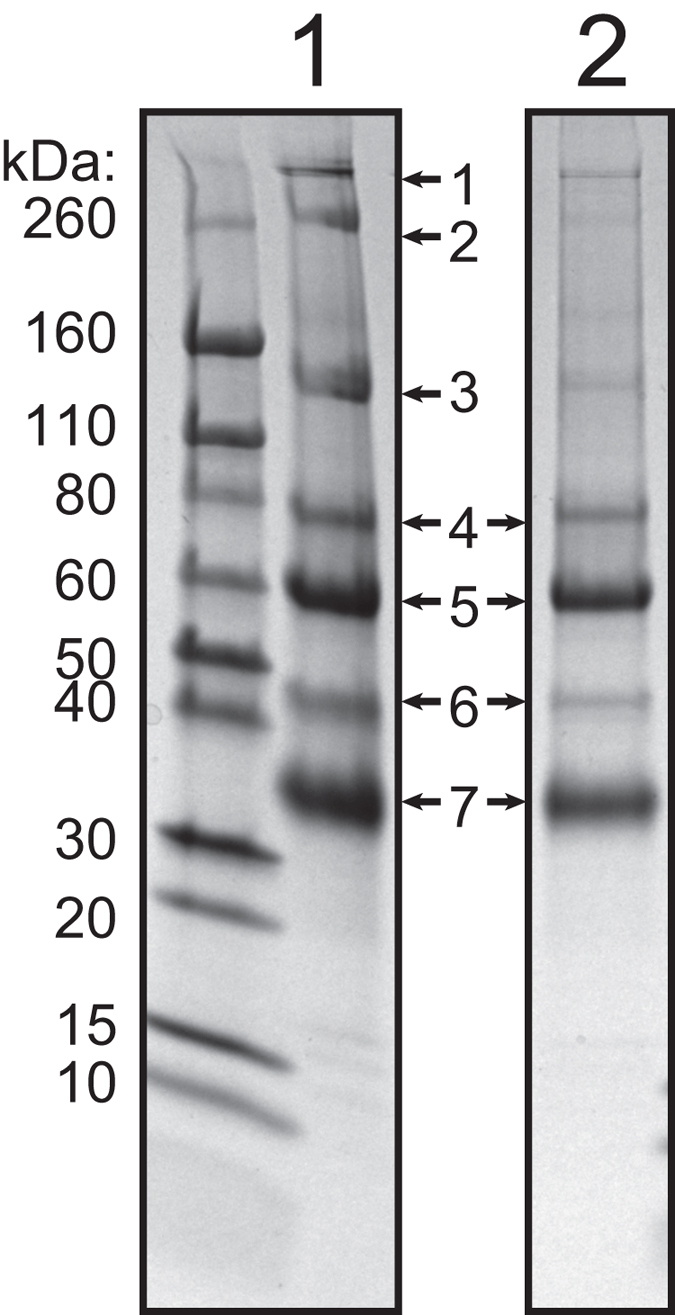
SDS-PAGE of crystal extractions from arm tip photophores. SDS-PAGE was used to analyse extracted crystal samples revealing seven prominent bands in preparation A (lane 1), and four prominent bands in preparation B (lane 2), all marked with arrows. Molecular weight marker sizes in kDa are provided on the left. Bands analysed using mass spectrometry from lane 1 (preparation A) are numbered 1 to 7.

Extracted crystal samples were analysed using SDS-PAGE. Two preparations are pictured in Fig. 3: preparation A (lane 1), in which seven bands were revealed, and preparation B (lane 2), which showed four prominent bands. Both samples clearly showed two bands of approximately 59 and 81 kDa, assumed to be equivalent to the bands observed by Hamanaka *et al*. (approximately 63 and 81 kDa), where the crystals were purified using sucrose gradient centrifugation^12^. Whereas Hamanaka *et al*. estimated the relative quantity of the 63 and 81 kDa proteins in the crystals from their SDS-PAGE gel to be 8:1, we estimated the stoichiometric ratio of the protein bands from our SDS-PAGE analyses to be about 4:1 to 5:1.

We identified the proteins present in the most prominent seven bands seen in preparation A. Bands of interest were excised from the gel and digested using trypsin. Masses of resultant peptides were measured using MALDI tandem Time-of-Flight mass spectrometry (MALDI TOF/TOF) and then searched against the combined squid transcriptome assembly translated into all possible reading frames. Proteins identified in this way are presented in Table 1 along with putative annotations and peptide search scores, number of peptide matches and % sequence coverage. Details of the mass spectrometry analysis, including peptides identified, the sequences of all matched transcripts and sequence coverage are given in Supplementary Tables S3 and S4. Annotated sequence homologs of the squid proteins were found using BLASTX^14^ and the Genbank protein sequence database (NCBI; http://www.ncbi.nlm.nih.gov).

Of primary interest are the proteins in bands 4 and 5, with similar molecular weights to the two protein bands identified by Hamanaka *et al*. Surprisingly, we identified not two, but three different proteins as the main constituents of these two bands (see Table 1). We will refer to these three proteins as wsluc1 (encoded by transcript 82699_c0_seq1), wsluc2 (81000_c2_seq2) and wsluc3 (83251_c0_seq1). Band 4 primarily consists of wsluc1 (17 peptide matches, Mascot score of 1374), predicted to be a 75.9 kDa protein. Band 5 consists mainly of two proteins: wsluc2 and wsluc3 (15 and 13 peptide matches and Mascot scores of 1592 and 914, respectively) with molecular weights of 62.3 and 61.7 kDa. Two peptides from wsluc3 were also detected in band 4 (Mascot score 115).

Wsluc1-3 appear to be paralogs, with all three containing motifs from the ANL superfamily of adenylating enzymes, which includes (and is named for) the Acyl-CoA synthetases, the NRPS adenylation domains, and the beetle (firefly) Luciferase enzymes^15^. Wsluc1 has a larger molecular weight than wsluc2 and 3 because of 105 additional amino acids on its N-terminus. The three proteins share 39% to 43% amino acid sequence identity over the full lengths of each protein except for the extra N-terminal residues in wsluc1.

There was one additional protein detected in both bands 4 and 5: an anion transporter family member protein (encoded by transcript 79083_c3_seq2 or 79083_c3_seq3), however only one peptide of this protein was detected in both bands (Mascot scores of 69 to 84).

The SDS-PAGE analysis also indicated that the crystal instability we observed was not a result of proteolysis. The molecular weights observed for bands 4 and 5 (about 81 and 59 kDa) are close enough to the predicted molecular weights of the identified proteins (75.9, 62.3 and 61.7 kDa) to suggest that these microcrystal proteins remain intact in the crystalline state. Since the sample run on this gel lane also contained dissolved crystals, it appears that the proteins remain intact in the soluble state as well - there are no smaller molecular weight bands present that indicate proteolysis occurs.

Nearly all of the remaining proteins identified in preparation A (found in bands 1, 2, 3, 6 and 7) appear to play roles in cellular structure (various collagens or beta actin), or signalling (cAMP-dependent protein kinase subunits, guanine nucleotide binding protein (G protein) subunits), possibly regulating light organ metabolism. One protein found in band 7, a 38.3 kDa protein encoded by transcript 52425_c0_seq1, had no homology with any sequences in the database and has unknown function.

### Search of the mantle transcriptome assembly for homologs of the crystal proteins

There has been very little investigation into either the eye or cutaneous photophores, presumably because they are small and more difficult to dissect; therefore it is not known if they use the same mechanism as the arm tip photophores to produce light. According to Teranishi and Shimomura “…Both types of luminescence probably involve an identical chemical mechanism because no example is known for the occurrence of two chemically different bioluminescence systems in one organism”^4^. However, the arm tip and cutaneous photophores are quite different in size, form and function, which may indicate some differences in bioluminescence at a biochemical level. The arm tip photophores produce very intense blue light, whereas the cutaneous photophores glow with a much lower intensity in either green or blue^1^. Light and electron micrographs of both types of photophore published by Okada in 1966^2^ revealed that the smaller cutaneous photophores also contain some “rodlets”, but these are much fewer in number and are a different shape to the crystals found in the arm tip organs. Okada described them as being “fusiform, 9–13 μ long and 2 μ wide at the widest median portion, and are not separated into blocks” [sic]. It remains unclear whether the bioluminescence of the cutaneous organs originates from these rodlet structures, and whether the rodlets are proteinaceous and crystalline.

To investigate the bioluminescence of cutaneous photophores, we searched the mantle transcriptome assembly for any proteins homologous to the crystal protein sequences from the arm tip organs. Using the tBLASTn algorithm within CLC Genomics Workbench (version 8.5.1; http://www.clcbio.com), searches revealed a single transcript (transcript c23316_g1_i1) encoding a protein with reasonable homology to any of the three crystal proteins, which we will refer to as wsluc4. This protein shares 83%, 45% and 38% amino acid sequence identity with wsluc2, wsluc3 and wsluc1, respectively, along the whole length of each protein except the extra 105 N-terminal residues in wsluc1. The next most similar full-length protein-encoding transcripts in the mantle assembly were only 19 to 20% identical with wsluc1–3.

When we ‘back-searched’ the combined transcriptome using wsluc4, the closest transcript found was 81000_c2_seq1, a variant of transcript 81000_c2_seq2 (wsluc2), which was not detected in the mass spectroscopy analysis. The protein encoded by 81000_c2_seq1 is 92% identical to wsluc4, and 89% identical to wsluc2. An alignment of these three proteins is provided in Supplementary Fig. S1.

It is unclear whether 81000_c2_seq1 and 81000_c2_seq2 result from either alternative splicing or paralogous genes^16^. At this point we cannot distinguish between the two types of variants because the full genome for the firefly squid, which has not yet been sequenced, is unavailable for reference. It is also unclear why wsluc4 does not have an identical equivalent in the combined assembly. It is possible that wsluc4 is a specific isoform from the mantle that is diluted when the various libraries are merged, but again, the full genome is required to clarify this issue.

### Sequence alignment and phylogenetic analysis of crystal proteins

To find out what sequence motifs the three crystal proteins (wsluc1–3) and their mantle homolog (wsluc4) share with known luciferase and luciferase-like proteins and other similar proteins, and the evolutionary relationships between these proteins, we carried out sequence alignments and a phylogenetic analysis. We searched for the closest homologs of the three crystal proteins (wsluc1–3) and their mantle homolog (wsluc4) among all known proteins in the non-redundant Genbank NCBI protein sequence database. We also looked for homologs for which functional information has been provided experimentally in the manually annotated and reviewed Swiss-Prot section of the UniProt Knowledgebase (http://www.uniprot.org/).

No close sequence homologs were found for the four squid proteins. The only hits from Genbank above 30% identity were for proteins from another member of the phylum Mollusca, *Octopus bimaculoides* (up to 40% identity). The closest homolog for which functional information has been provided experimentally is human acyl-CoA synthetase family member 2, also known as ACSF2 (24% to 26% identity). ACSF2 activates medium chain fatty acids by forming a thioester with coenzyme A^17^. Firefly luciferases did not feature in the top ranked hits in any of the searches.

**Figure 4:**
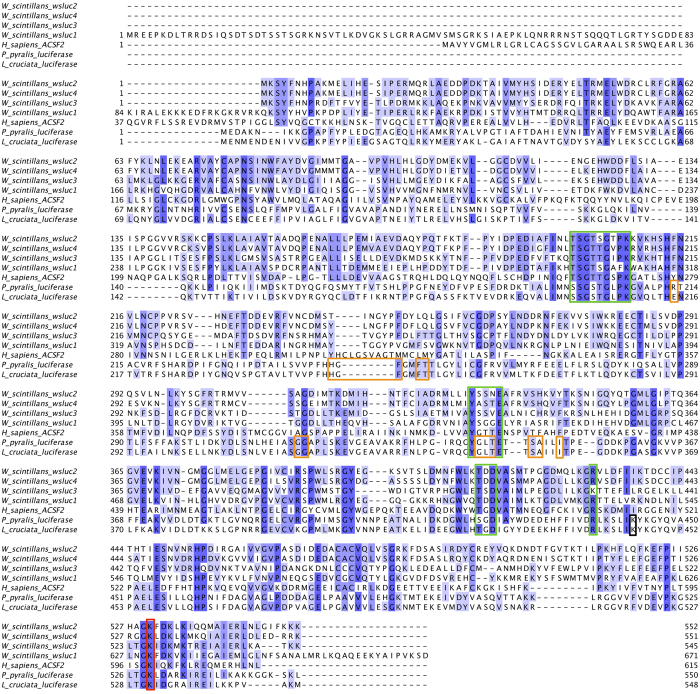
Alignment of wsluc1-4 proteins from *W. scintillans* with luciferase proteins from the fireflies *P. pyralis* and *L. cruciata* and the human ACSF2. Residues are coloured according to the percentage of the residues in each column that agree with the consensus sequence (the darker the blue, the higher the percentage agreement). Green boxes indicate positions of ATP-binding motifs conserved throughout the ANL superfamily^15^, and orange boxes indicate residues that bind luciferin in the firefly luciferase^18^,^19^. The red box indicates the lysine residue that plays a key catalytic role in the adenylation half reaction throughout the ANL superfamily^15^, and the black box indicates the lysine residue that plays a key role in the oxidation (light-producing) half reaction in firefly luciferase^20^.

An alignment of the four squid proteins with two firefly luciferases from *Photinus pyralis* (North American firefly) and *Luciola cruciata* (Japanese firefly) and human ACSF2 is provided in Fig. 4, and demonstrates that all four squid proteins are members of the ANL superfamily of adenylating enzymes^15^. The enzymes in this superfamily catalyse a wide range of different overall reactions, but all carry out two partial reactions, the first being an activation step where a carboxylate substrate is adenylated using ATP. They feature two domains; the substrate and ATP bind in a pocket located within the large N-terminal domain, which is capped by the smaller C-terminal domain. The C-terminal domain can rotate by 140° to present opposing faces to the active site for the different partial reactions^15^. The squid proteins contain residues conserved throughout the ANL superfamily, particularly ATP-binding residues and the lysine residue that plays a key catalytic role in the adenylation half reaction (indicated by green and red boxes, respectively, in Fig. 4). Residues that bind luciferin^18^,^19^ and the lysine residue that plays a key role in the oxidation (light-producing) half reaction^20^ in the firefly luciferase enzymes are apparently not well conserved in the squid proteins. However, it is possible that a lysine (if a lysine is required for the squid enzyme second half reaction) is provided from an adjacent helix or strand, so is not visible in a sequence alignment.

We carried out a phylogenetic analysis to see how the squid crystal proteins might be grouped relative to known firefly luciferase proteins and other non-luminescent members of the ANL superfamily. An alignment was made including the firefly luciferase sequences from *L. cruciata* and *P. pyralis*, as well as the non-luminescent luciferase-like homolog from *L. cruciata*^21^,^22^, firefly luciferase-like proteins from non-luminescent organisms (*Drosophila melanogaster* CG4830 and pdgy^21^,^23^; *Aedes aegypti*; *Tenebrio molitor*^24^), firefly luciferase-like proteins from non-luminescent organisms with weak luciferase activity (*D. melanogaster* CG6178^25^; *Zophobas morio* luciferase-like^26^), and candidate luciferases from the New Zealand glowworm, *Arachnocampa luminosa* (64201_seq1, 64201_seq2, 62762^27^). Three more sequences were added from the Mollusca phylum: *O. bimaculoides* accessions KOF97006, XP_014779992 and XP_014790470. Proteins from three members of the Chordata phylum were used as an outgroup: human and mouse acyl CoA synthetase proteins and a homolog from the lancelet *Branchiostoma floridae*. After automatic curation, the final amino acid alignment was composed of 277 residues, from which a Bayesian phylogenetic tree was calculated.

**Figure 5:**
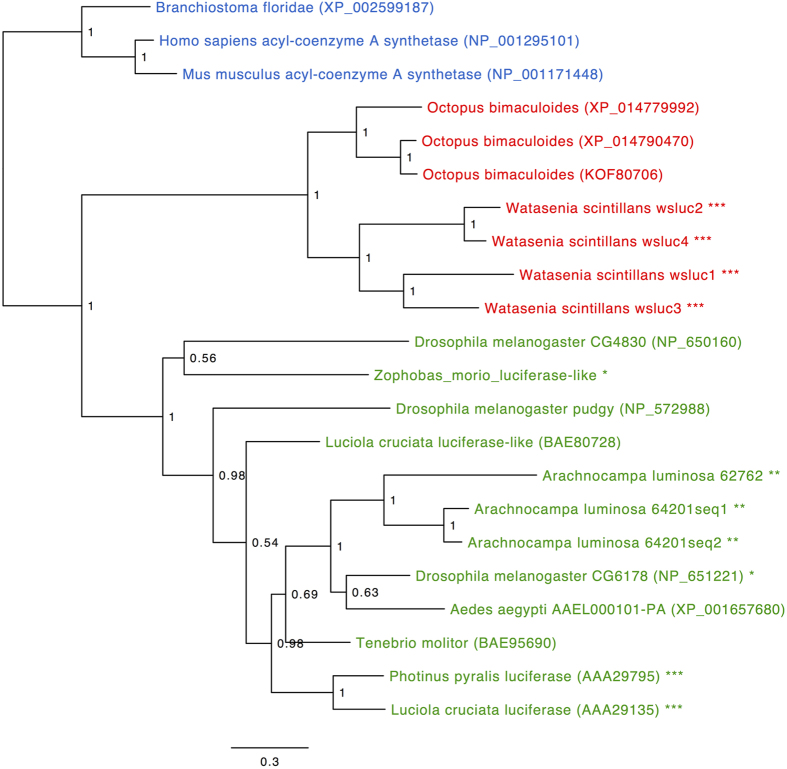
Phylogenetic tree of squid crystal proteins and homologous sequences from the ANL superfamily of enzymes. Branch lengths are proportional to the number of substitutions per site (see scale bar). Numbers at each internal node represent Bayesian posterior probabilities. GenBank accession numbers for either protein or nucleotide sequences where available are shown in parentheses. Taxa are coloured according to phyla: Chordata blue, Arthropoda green and Mollusca red. ***Luciferases from bioluminescent creatures; **candidate luciferases from bioluminescent creatures; *enzymes that produce light but are from non-luminescent creatures.

Three main clades can be seen in the phylogenetic analysis (Fig. 5). Proteins that are known luciferase enzymes or are candidate luciferase enzymes did not group together. Instead the sequences formed groups according to the phyla of the creatures they belong to: the squid crystal proteins were grouped with the octopus sequences (Mollusca), and the insect (Arthropoda) and Chordata protein sequences each formed separate clades. Within the Arthropoda clade it can be seen that the various insect luciferases and luciferase-like proteins are spread throughout and are not always grouped according to species, which is a reflection of complex gene duplication events and functional divergence that has occurred in this protein family in bioluminescent beetles and other insects^28^. The established evolutionary relationships between animal phyla are reviewed elsewhere^29^.

### Functional annotation of the most highly expressed transcripts in the arm tip and mantle samples

We used RNAseq to identify the most abundant transcripts in the two different tissues, and then annotated the top 500 most expressed in each tissue type. First, reads were mapped from each of the six sequence libraries separately onto the combined transcriptome assembly, since there is no reference genome available for *W. scintillans*, generating fragments per kilobase of transcript per million mapped reads (FPKM) values for each transcript in each library. After calculating the average FPKM for every transcript across the libraries for the arm tip and mantle tissues separately, the 500 transcripts with highest FPKM values were selected for each tissue type. We used BLASTX matches from the non-redundant database at the NCBI to assign Gene Ontology (GO) terms to the two subsets of most abundant transcripts. 264 and 322 transcripts were assigned GO terms for the arm tip and mantle tissue transcripts, respectively. Supplementary Tables S5 and S6 list the 500 most abundant transcripts for arm tip and mantle tissue samples, respectively, along with average FPKM values, annotations from BLAST results and GO terms.

We also carried out a gene set enrichment analysis for each of these sets of transcripts to detect sets of genes that have a common behaviour or annotation. The total numbers of significant terms (p-value > 0.05) identified for the highest abundance arm tip and mantle transcripts were 86 and 44, respectively, and these terms are listed in Supplementary Tables S7 and S8. GO terms associated with mitochondria, oxidative phosphorylation and ATP synthesis were prominent in the enrichment analysis for the both tissues. This may be due to the high requirement for ATP in the photophore tissue.

The three crystal proteins and their mantle homolog did not feature in the lists of most abundant transcripts. The average FPKM levels for each of the transcripts encoding these proteins and their rankings among the overall lists of transcripts ordered by expression level are detailed in Table 2. Wsluc1–3 have average FPKM values of 8.3 to 11.7 (ranked at about the top 5400 to 7700^th^), and wsluc4 an average value of 2.8 (around the top 19500^th^ of the most abundant transcripts in arm tip tissue); in mantle tissue they have average FPKM values of 0.02 to 0.84 (ranked from about the top 14000^th^ to the top 69000^th^ transcripts). In comparison, the most abundant transcripts have average FPKM values of 11,600 (arm tip tissue) and 28,500 (mantle tissue; see Supplementary Tables S5 and S6). This does not necessarily mean that these transcripts are not highly expressed in the photophores themselves. The tissue used to prepare cDNA for the sequencing libraries included both photophores and non-luminous tissues. Therefore the relatively lower levels of wsluc1–4 transcripts may be due to the dilution of the bioluminescence-related transcripts by the transcripts from the non-luminous tissue.

## Discussion

**Figure 6:**
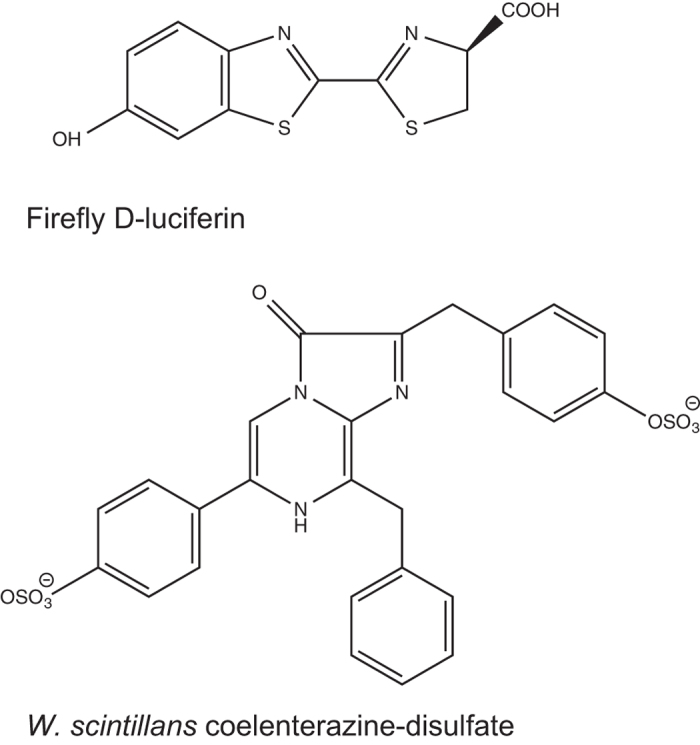
Luciferin substrates of *W. scintillans* and firefly bioluminescence reactions.

In this study we have identified three closely related proteins that comprise the bioluminescent microcrystals of the Japanese firefly squid arm tip photophores (wsluc1–3), and a homolog in the squid mantle (wsluc4). BLAST searches showed that all four proteins are clearly members of the ANL superfamily of enzymes, which use ATP to adenylate and activate substrates for further catalysis. The sequences have 19% to 21% sequence identity with the luciferase from the firefly *P. pyralis*, which also uses ATP to adenylate its luciferin substrate, although the closest homologs to the squid crystal proteins found in the database searches were not firefly luciferases but sequences annotated as acyl-CoA synthetases. Additionally, it is well established that *W. scintillans* requires ATP as well as coelenterazine disulfate to produce light. Therefore it is highly likely that at least one of wsluc1–4 is responsible for bioluminescence in *W. scintillans*. Tsuji proposed a reaction mechanism for the production of light by the squid based on investigations into the biochemical basis of *W. scintillans* bioluminescence^3^,^9^, where coelenterazine disulfate is first adenylated using ATP, then reacted with oxygen to form an unstable dioxetanone intermediate, which spontaneously decomposes producing light. The identification of the squid crystal proteins as members of the ANL family and potential luciferase enzymes supports the overall idea of this scheme: a single ANL family enzyme could potentially catalyse both the adenylation and oxidation reactions, as occurs in firefly luciferase. The differences between the substrate-binding residues of the firefly luciferases and the equivalent residues of the squid proteins (Fig. 4) will most likely reflect the differences in the structures of their substrates (firefly D-luciferin vs coelenterazine-disufate; Fig. 6).

Bioluminescence has evolved independently at least 40 times across extant organisms. As a result, luciferase enzymes characterised so far have extremely varied structures, mechanisms and substrate specificities^30^,^31^,^32^. It has been thought that each luciferase enzyme from each independently evolved bioluminescent system was unique, with no sequence similarity between enzymes from different lineages^30^,^33^. Luciferin substrate structures also vary significantly, for example, firefly luciferin is a unique benzothiazole, bacterial luciferin is a long-chain aldehyde, and dinoflagellate luciferin is a tetrapyrrole similar to chlorophyll. However, sometimes the same luciferin substrate has been independently co-opted into bioluminescence in unrelated organisms. For example, the shrimp *Oplophorus*, and the cnidarians *Aequorea* and *Renilla*, among others, all use coelenterazine or a modified form of coelenterazine as their luciferin, even though they use luciferases that are unrelated in structure and sequence.

It now appears that the ‘co-opting’ of similar or the same luciferin substrates into bioluminescence can also occur with luciferase enzymes as well. There is already evidence that ANL superfamily enzymes have evolved the ability to produce light at least twice in insects. The firefly (beetle) luciferases evolved from non-luminescent acyl-CoA synthetases^22^,^34^,^35^; indeed, acyl-CoA synthetases from two nonluminous insects can bioluminesce in the presence of firefly luciferin substrate or a synthetic analog^25^,^26^ and firefly luciferase is a fully functional fatty acid CoA synthetase^36^. Further, we recently identified three candidate luciferases in another insect, the Dipteran New Zealand glowworm, *Arachnocampa luminosa*, all of which are members of the ANL superfamily and share sequence homology with acyl-CoA enzymes^27^. Now that the *W. scintillans* crystal proteins have been identified as ANL superfamily members as well, there is increasing evidence that a convergence of bioluminescent function can occur in these enzymes.

There are three possible scenarios that explain why ANL enzymes are repeatedly found to be luciferases in phylogenetically diverse creatures:

1. ANL proteins evolved bioluminescence in multiple, unrelated, independent events in different creatures.
2. An ANL protein evolved bioluminescence in the last common ancestor of the firefly squid, fireflies and *A. luminosa*, and this gene was inherited by all of these creatures, but was either not inherited by or lost bioluminescent ability in their numerous other descendent creatures.
3. An ANL protein evolved bioluminescence in one event and was passed between firefly squid, fireflies and *A. luminosa* by a horizontal gene transfer mechanism.

Our phylogenetic analysis provides evidence that the squid crystal proteins are more closely related to non-bioluminescent ANL family proteins from other Molluscs than other bioluminescent ANL family proteins. Option one is the more parsimonious of the three, especially since the substrates used by fireflies and the firefly squid are very different. Therefore, ANL superfamily enzymes in phylogenetically distant species can, and may have a propensity to, independently evolve the ability to catalyse bioluminescence, even with different substrates.

Why the ANL enzymes have evolved a step further in *W. scintillans* than the luciferases found in insects and developed the ability to form crystals is so far unknown, however, Hamanka *et al*. suggest that the dense packing of the bioluminescent system in a crystal structure may enable *W. scintillans* to produce particularly intense light^12^.

It is unclear how the wsluc1–3 proteins might interact to form the glowing squid crystals, or to catalyse bioluminescence. We propose that they form a complex that crystallises inside the squid photophore, where one, two or possibly all three of the proteins have bioluminescent catalytic activity. Although firefly (*P. pyralis*) luciferase is monomeric^37^, other members of the ANL superfamily are sometimes known to form multimeric assemblies. Some form homodimers, including an *o*-succinylbenzoyl-CoA synthetase from *Bacillus subtilis*^38^, 4-chlorobenzoate: CoA ligase from *Alcaligenes sp. AL3007*^39^, long chain fatty acyl-CoA synthetase from *Thermus thermophilus*^40^, acyl coenzyme A synthetase from *Escherichia coli*^41^, and the human acyl-CoA synthetase long-chain member 6^42^. A homotrimer has also been reported: *Saccharomyces cere*v*isiae* acetyl-coenzyme A synthetase forms a trimer where residues from the large N-terminal domains of three monomers bind together; the small C-terminal domains located at the periphery^43^. It is possible the three squid crystal proteins may assemble into a complex that resembles the reported trimer, which then in turn assembles into the crystal lattice. The crystal lattice may need to allow the C-terminal domain of the catalytically active peptide(s) to rotate, facilitating two different partial reactions, as occurs in other ANL family enzymes. Alternatively, if this large motion was not able to occur in the crystal lattice, it may be that within the crystalline complex different proteins may stay locked in different conformations, each carrying out only one of the two partial reactions. It would be interesting to establish whether the bioluminescence can only occur when the crystal proteins are in crystalline form. The unsuccessful attempts at solubilisation of the active luciferase by Teranishi *et al*.^4^ suggest that this may be the case.

Further research is needed to elucidate the exact composition of the bioluminescent crystal complex. Our SDS-PAGE and mass spectrometry analyses suggest that all three wsluc1–3 proteins are present in the bioluminescent crystals. These analyses along with SDS-PAGE analysis from Hamanaka *et al*. suggest approximate ratios of 62 kDa protein to 76 kDa protein that vary from 4:1 to 8:1. However, none of these analyses carried out so far provide us with any certainty what ratio these proteins might exist in within a crystal forming complex.

It is clear from the alignment in Fig. 4 that the N-terminal domain of wsluc1 is not present in wsluc2–4 or firefly luciferase. Although no peptides in the mass spectrometry analysis of Band 4 display matched this 105 amino acid domain, it is still highly likely that the predicted N-terminal domain is present in the protein and is not an artefact of assembly because of three reasons. (1) The total predicted mass of wsluc1 was comparable with the band size on SDS-PAGE. (2) When carrying out a BLAST search of the the NCBI database, a homologous predicted octopus protein, *O. bimaculoides* KOF80706 (unknown function), was found that has an N-terminal region with homology to that of wsluc1 (23% identity over the 105 amino acids of wsluc1 and the 142 N-terminal amino acids of the octopus sequence). (3) Sequencing reads mapped onto the 82699_c0_seq1 (wsluc1) transcript (3985 nucleotides long) covered the nucleotides encoding the N-terminal domain (183 to 497) really well: the mean coverage of these nucleotides was 70 reads (standard deviation of 15.6).

It is unclear what function the wsluc1 N-terminal domain has. Other than the *O. bimaculoides* protein, no other matches were found between the domain and any other sequences in the NCBI database. The N-terminus of wsluc1 is not predicted to contain transmembrane helices, but it is predicted to be intrinsically unstructured and is rich in protein binding sites according to the PredictProtein compilation of algorithms^44^. No signal peptides were found using the SignalP 4.1 Server (http://www.cbs.dtu.dk/services/SignalP/)^45^. However, it might still function as a signal peptide, targeting the protein to a particular cellular compartment, although one might expect to see it on all three proteins, unless all three proteins form a complex first, which is then translocated as a single entity by the peptide. It is also possible that the sequence may facilitate crystal formation.

A single homolog of the squid crystal proteins was found in the mantle transcript assembly, wsluc4, which suggests that bioluminescence in the cutaneous photophores might operate differently than in the arm tips: either they produce light using one rather than three ANL superfamily proteins, or they use entirely different proteins that have not yet been identified.

Future research to verify the role of the wsluc1–4 proteins in *W. scintillans* bioluminescence is required, and will include producing the proteins recombinantly, and assaying them for activity using coelenterazine disulphate, ideally in both a soluble state and as crystals.

In conclusion, we have identified three different but homologous proteins from the bioluminescent microcrystals of the Japanese firefly squid (wsluc1–3), and a close homolog in the squid mantle (wsluc4), which are all members of the ANL superfamily of adenylating enzymes. Further research is required to confirm the role of these crystal proteins in *W. scintillans* bioluminescence and determine how they might interact together to form catalytically active crystals. Nonetheless, it appears that the firefly squid bioluminescent enzyme has evolved from the same superfamily of enzymes as the firefly (beetle) luciferase enzymes, even though they use different luciferin substrates. This research suggests that members of the ANL enzyme superfamily have characteristics that enable them to evolve the ability to produce light, even with entirely different substrates and in phylogenetically distant organisms such as insects and cephalopods. Therefore, whenever a bioluminescent system is shown to require ATP, researchers should consider the possibility that the luciferase enzyme involved is also a member of the ANL superfamily of adenylating enzymes.

## Methods

### Sample collection, RNA and crystal extraction

Squid dissection in this project was carried out in Japan; ethical approval was not required for the project within the Japanese regulatory framework. Nonetheless, the University of Otago Animal Ethics Committee was advised of the use of squid in this research, ethical and welfare considerations were taken into account and recommended procedures were followed^46^. *W. scintillans* purchased from local fishermen were obtained from Uozu Aquarium, Toyama Prefecture, Japan. They were either stored at 4 °C and dissected within 12 hours, or kept alive in tanks for up to two days before being euthanised and dissected. The arm light organs continued to glow for several minutes after dissection.

For cDNA sequencing, four arm tips, each with three large photophores (1.3 to 2.4 mg), and two small sections of mantle (68 and 132 mg) were cut from six different squid. Samples were soaked in RNA preservation solution (EDTA 13.3 mM, sodium citrate 16.67 mM, ammonium sulfate 3.53 M, pH 5.2) overnight, solution drained off, and samples stored at −20 or −80 °C. Tissues were homogenised with TRIzol^®^ Reagent (Invitrogen/ThermoFisher Scientific) using a glass dounce homogeniser. UltraPure^TM^ (Phenol:Chloroform:Isoamyl Alcohol; Invitrogen/ThermoFisher Scientific) was used to extract total RNA, which was then purified further using the RNeasy Kit (Qiagen). An RNA chip (Bioanalyzer 2100, Agilent Technologies) was used to quantify and assess integrity for each RNA sample.

Crystals were extracted from arm tip samples first by dissecting photophores from surrounding arm tip tissue using a scalpel and tweezers. Photophores were homogenised in phosphate-buffered saline (PBS) with 40% sucrose using a glass dounce homogeniser and filtered through a 11μm pore nylon membrane with a syringe. The crystals were then washed by adding more PBS with 40% sucrose, centrifuging at 8 000 x*g* in a microfuge for 10 minutes and discarding the supernantant, for two cycles. The final pellet containing the crystals was resuspended in PBS with 40% sucrose, and the sample stored at 4 °C.

### cDNA library construction, sequencing and quality control and *de novo* assembly

The six total RNA samples (25 μl each at 41 to 2016 ng/μl), each with an RIN of over 6 (arm tip samples) or 4 (mantle samples) were delivered to the Otago Genomics and Bioinformatics Facility for mRNA isolation using oligo-dT magnetic beads, and cDNA library construction using the Illumina TruSeq Stranded mRNA Sample Preparation Kit. The Illumina HiSeq-2000 machine was used for sequencing, with each sample run on one eighth of a sequencing lane, generating 100 bp paired-end reads. The TruSeq stranded mRNA library provided information on strand origin (from which of the two DNA strands a given RNA transcript was derived) which can increase the percentage of reads that can be aligned, and therefore improve transcript reconstruction compared with non-strand specific data^16^.

Adaptor sequences were trimmed from reads using fastq-mcf^47^, and bases with low quality phred scores trimmed (cut-off score of Q20). Adapter and quality trimmed reads less than 50 nucleotides in length were discarded using the SolexaQA package^48^, and reads were assessed for quality using FASTQC (http://www.bioinformatics.bbsrc.ac.uk/projects/fastqc).

Reads were assembled using the Trinity software package^49^ with parameters adjusted for Illumina stranded paired end sequencing (i.e. –left and –right for both R1 and R2 using –SS_lib_type RF for the stranded library type).

### Protein identification

#### SDS-PAGE and mass spectrometry analyses

Extracted crystals were prepared for analysis by SDS-PAGE by boiling with SDS-PAGE sample buffer containing β-mercaptoethanol. Samples were then run on Mini-PROTEAN^®^ TGX^TM^ 4–20% polyacrylamide precast SDS-PAGE gels (BioRad) and stained using Coomassie blue. Gels were scanned using a Gel Doc^TM^ XR+ system (BioRad), and molecular weight and relative quantity estimates for gel bands were calculated using Image Lab software version 5.0 (BioRad).

Each band was excised from the gel, subjected to tryptic digestion, and peptides analysed on a 4800 MALDI tandem Time-of-Flight Analyzer (MALDI TOF/TOF, Applied Biosystems, MA) at the Centre for Protein Research (University of Otago, Dunedin, New Zealand). The resulting peptide mass data were searched against the combined squid transcriptome assembly translated into all possible reading frames, using the Mascot search engine (http://www.matrixscience.com).

#### Alignment and phylogenetic analysis

Multiple sequence alignments were performed using the MUSCLE tool^50^ on the EMBL-EBI web server (http://www.ebi.ac.uk/Tools/msa/muscle/), and visualised using Jalview (http://www.jalview.org). The alignment for phylogenetic analysis was then edited to eliminate poorly aligned positions and divergent regions using the Gblocksweb server^51^ (http://molevol.cmima.csic.es/castresana/Gblocks_server.html). Phylogenetic analysis was performed using MrBayes 3.2.3^52^ on the Phylogeny.fr web service server (http://www.phylogeny.fr/one_task.cgi?task_type=mrbayes) under a WAG substitution model (selected using ProTest 3.2^53^), run for 10,000 generations. Trees were sampled every 10 generations; the final consensus tree was calculated after the first 250 trees sampled were discarded. The phylogenetic tree was visualised using FigTree 1.4.2 (http://tree.bio.ed.ac.uk/software/figtree/).

#### Read mapping, measurement of gene expression and functional annotation

Reads from each of the six samples were mapped separately onto the combined assembly using Bowtie 2^54^, and transcript abundance in FPKM (fragments per kilobase of transcript per million fragments mapped) was calculated for each sample using the RSEM package^55^. We then calculated the average FPKM for every transcript across the arm tip samples and across the mantle samples, then ranked both lists according to average FPKM values. The 500 most abundant transcripts for each tissue type were annotated by identifying similar annotated proteins where function could be inferred using Blast2GO v3.1 (http://www.blast2go.com)^56^. BLASTX searches^14^ against the GenBank non-redundant database at the NCBI were carried out with an E-value cut-off of 10^−3^, and the top 20 hits were recorded for each transcript. Blast2GO assigned GO annotations to transcripts using the BLASTX results. A gene set enrichment analysis was carried out for each set of annotated transcripts, ranked according to average FPKM values, using the FatiScan/Logistic Model Gene Set Enrichment module^57^,^58^,^59^ from Babelomics 5^60^ (http://babelomics.bioinfo.cipf.es/).

### Data availability

Raw sequence data from this experiment were submitted in FASTQ format to the NCBI Sequence Read Archive (SRA) database (accessions SRR2960126, SRR2960127, SRR2960128, SRR2960130, and SRR2960131) and are also accessible through the BioProject accession PRJNA303268 (http://www.ncbi. nlm.nih.gov/bioproject/PRJNA303268). The three transcriptome assemblies have been deposited at the NCBI Transcriptome Shotgun Assembly (TSA) database and are available at the DNA DataBank of Japan (DDBJ), the European Molecular Biology Laboratory (EMBL), and GenBank at NCBI under the accessions GEDW00000000 (arm tip), GEDX00000000 (mantle) and GEDZ00000000 (combined mantle and arm tip).

## Additional Information

**How to cite this article**: Gimenez, G. *et al*. Mass spectrometry analysis and transcriptome sequencing reveal glowing squid crystal proteins are in the same superfamily as firefly luciferase. *Sci. Rep*. **6**, 27638; doi: 10.1038/srep27638 (2016).

## Supplementary Material

Supplementary Information

Supplementary Table S3–S6

## Figures and Tables

**Figure 1:**
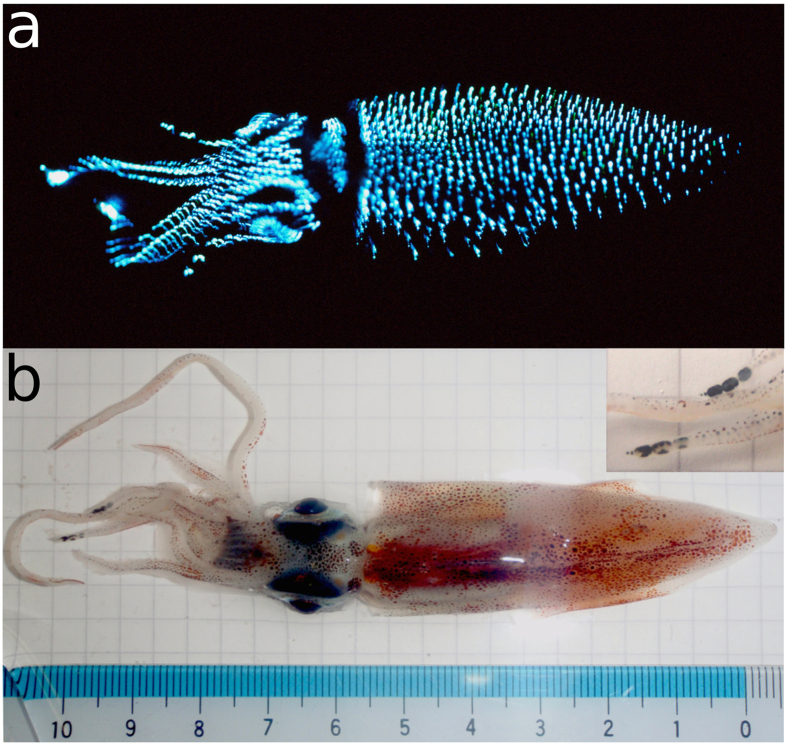
Photographs of *W. scintillans*. Ventral view illuminated by (**a**) its own light (taken by Osamu Inamura), and (**b**) by an external light source (taken by Neil Paterson). Inset: closer view of arm tip light organs.

**Table 1:** Summary of proteins from crystal protein extraction identified using MALDI TOF/TOF mass spectrometry analysis.

SDS-PAGE protein band		Protein identification			Mascot (MALDI TOF/TOF)		
Number	Molecular weight (kDa)	Transcript number	Putative Annotation	Molecular weight (kDa)	Score	Number of peptide matches	% sequence coverage
1	271	70085_c0_seq1/ 70085_c0_seq2	collagen alpha chain	126.9 or 127.9	75	2	2.0
2	244	70085_c0_seq1/ 70085_c0_seq2	collagen alpha chain	126.9 or 127.9	115	2	1.8
		52532_c0_seq1	collagen alpha chain	142.2	76	1	1.0
3	137	52532_c0_seq1	collagen alpha chain	142.2	233	2	2.7
		70085_c0_seq1/ 70085_c0_seq2	collagen alpha chain	126.9 or 127.9	91	2	1.8
**4**	**81**	**82699_c0_seq1**	**acyl-coA synthetase family member**	**75.9**	**1374**	**17**	**35.3**
		83251_c0_seq1	acyl-coA synthetase family member	61.7	115	2	4.4
		79083_c3_seq2/ 79083_c3_seq3	anion transporter family member	80.6	69	1	2.3
**5**	**59**	**81000_c2_seq2**	**acyl-coA synthetase family member**	**62.3**	**1592**	**15**	**45.7**
		**83251_c0_seq1**	**acyl-coA synthetase family member**	**61.7**	**914**	**13**	**29.2**
		79083_c3_seq2/ 79083_c3_seq3	anion transporter family member	80.6	84	1	2.3
6	40	77978_c0_seq4	beta actin	41.7	813	9	34.4
		64040_c2_seq3	guanine nucleotide binding protein alpha subunit	44.6	649	10	28.1
		69530_c0_seq1/ 69530_c0_seq2	cAMP-dependent protein kinase type II regulatory subunit	33.7 or 43.9	390	4	12.1–15.8
		85510_c0_seq1/ 85510_c0_seq2/ 85510_c0_seq3	cAMP-dependent protein kinase catalytic subunit	40.4, 40.5 or 49.7	175	2	7.2–9.0
7	28	52425_c0_seq1	no identification	38.3	670	9	29.7
		58459_c0_seq1	guanine nucleotide binding protein beta subunit	37.4	384	4	12.3

Proteins that are the predominant constituents of the two bands associated with the luminescent microcrystals are highlighted in bold.

**Table 2:** Average FPKM levels and abundance rankings for crystal protein-encoding transcripts.

Transcript number (protein name)	Arm tip tissue			Mantle tissue		
	Average FPKM	Standard deviation	Ranking	Average FPKM	Standard deviation	Ranking
82699_c0_seq1 (wsluc1)	8.4	5.3	7563	0.02	0.02	68966
81000_c2_seq2 (wsluc2)	11.7	8.0	5392	0.32	0.25	26800
83251_c0_seq1 (wsluc3)	8.3	5.5	7685	0.30	0.09	28126
81000_c2_seq1 (wsluc4)	2.8	2.3	19516	0.84	0.39	14013

The average FPKM levels for each of the squid crystal protein-encoding transcripts and their mantle homolog, and their rankings among the overall lists of transcripts ordered by expression level.
