# Supplementary material for: Mass spectrometry analysis and transcriptome sequencing reveal glowing squid crystal proteins are in the same superfamily as firefly luciferase
Source: Sci Rep. 2016 Jun 9;6:27638. doi: 10.1038/srep27638 (PMC4899746; doi:10.1038/srep27638)
Supplement: Supplementary Information [file srep27638-s1.pdf]

Mass spectrometry analysis and transcriptome sequencing reveal glowing squid crystal proteins are in the same superfamily as firefly luciferase

Gregory Gimenez[1], Peter Metcalf[2], Neil G. Paterson[3], Miriam L Sharpe[4]*

[1]Otago Genomics & Bioinformatics Facility, University of Otago, Dunedin, New Zealand
[2]School of Biological Sciences, University of Auckland, Auckland, New Zealand
[3]Diamond Light Source, Harwell Science and Innovation Campus, Didcot OX11 0DE, UK
[4]Department of Biochemistry, University of Otago, Dunedin, New Zealand
*Corresponding author: miriam.sharpe@otago.ac.nz

Additional file 1: Supplementary Figure S1 and Tables S1, S2, S7 and S8

Supplementary Figure S1. Alignment of wsluc2, wsluc4 and 81000_c2_seq1

Residues are coloured according to the percentage of the residues in each column that agree with the consensus sequence (the darker the blue, the higher the percentage agreement).

```
81000_c2_seq2_(wsluc2)    1 MKSYFNHPAKMELIHESIPERMQRLAEDDPDKTAIVMYHSIDERYELTRME 51
c23316_g1_i1_(wsluc4)     1 MKSYFNHPAKMELIHESIPERMQRLAEDDPDKTAIVMYHSIDERYELTRME 51
81000_c2_seq1             1 MKSYFNHPAKMELIHESIPERMQRLAEDDPDKTAIVMYHSIDERYELTRME 51

81000_c2_seq2_(wsluc2)   52 LWDRCLRFGRAFYKLNLEKEARVAYCAPNSINWFAYDVGIMMTGAVPVHLH 102
c23316_g1_i1_(wsluc4)    52 LWDRCLRFGRAFYKLNLEKEARVAYCAPNSINWFAYDVGMMMTGAVPVHLH 102
81000_c2_seq1            52 LWDRCLRFARAFFKLGLKKDARVAYCAPNSINWFAYDVGMMMTGAVPVHLH 102

81000_c2_seq2_(wsluc2)  103 LGDYDMEKVLGGCDVVLIENGEHWDDFLSIAEISPGGVVRSKKCPSLKLAI 153
c23316_g1_i1_(wsluc4)   103 LGDYDIETVLDGCDVVVIELKEHWDDFLAIAEILPGGVVRCKSVPSLKLAV 153
81000_c2_seq1           103 LGDYDIETVLDGCDVVVIELKEHWDDFLAIAEILPGGVVRCKSVPSLKLAV 153

81000_c2_seq2_(wsluc2)  154 AVTAADQPENALLLPEMIAEVDAQYPQTFKTFPYIDPEDIAFINLTSGTSG 204
c23316_g1_i1_(wsluc4)   154 AVTAVDQPENALLLPEMVAEVDAQYPQTFKPFPYIDPEDIGFINLTSGTTG 204
81000_c2_seq1           154 AVTAVDQPENALLLPEMIAEVDAQYPQTFKTFPYIDPEDIAFINLTSGTSG 204

81000_c2_seq2_(wsluc2)  205 TPKKVKHSHFNVLNCPPVRSVHNEFTDDEVRFVNCDMSTINGYPFDYLQLG 255
c23316_g1_i1_(wsluc4)   205 VPKRVRHSHFNVLNCPPVRSVHDEFTDDEVRFVNCDMSYLNGFPFDFLQLG 255
81000_c2_seq1           205 TPKKVKHSHFNVLNCPPVRSVHNEFTDDEVRFVNCDMSTINGYPFDYLQLG 255

81000_c2_seq2_(wsluc2)  256 SIFVCGDPSYLNDDRNFEKVVSIWKREECTILSVDPQSVLNLKYSGFRTRM 306
c23316_g1_i1_(wsluc4)   256 SVFVCGDPAYLNDPKNFEKIVSIWKKEECTMLSVDPESVKNLKYSGFRTRM 306
81000_c2_seq1           256 SIFVCGDPSYLNDDRNFEKVVSIWKREECTILSVDPQSVLNLKYSGFRTRM 306

81000_c2_seq2_(wsluc2)  307 VVSAGDIMTKDMIHNTFCIADRMLLIYSSNEAFRVSHKVYTKSNIGQYQTG 357
c23316_g1_i1_(wsluc4)   307 CVSSGEIMTKDMIHNTFCIADRMLMIYASTEAFRVSHQVFTKSNIGQYLPG 357
81000_c2_seq1           307 VVSAGDIMTKDMIHNTFCIADRMLLIYSSNEAFRVSHKVYTKSNIGQYQTG 357

81000_c2_seq2_(wsluc2)  358 MLGIPTQGVEVKIVNGMGGLMELGEPGIVCIRSPWLSRGYEGKSVTSLDMN 408
c23316_g1_i1_(wsluc4)   358 MLGLPTQGVEVKIVNGMGGLMELGEPGILSVRSPWLSRGYDGASSSSLDYN 408
81000_c2_seq1           358 MLGIPTQGVEVKIVNGMGGLMELGEPGIVCIRSPWLSRGYEGKSVTSLDMN 408

81000_c2_seq2_(wsluc2)  409 FWLKTDDVASMTPGGDMQLKGRVLDFIIKTDCCIPTHTIESNVNRHPDIRG 459
c23316_g1_i1_(wsluc4)   409 FWLKTDDVASMMPAGDLLLKGRVSDFIIKSDCCIPSATIESNVDRHPDVKG 459
81000_c2_seq1           409 FWLKTDDVASMMPAGDLLLKGRVSDFIIKSDCCIPSATIESNVDRHPDVKG 459

81000_c2_seq2_(wsluc2)  460 AIVVGVPASDIDEDACACVQLVSGRKFDSASIRDYCREYVQKDNDTFGVTK 510
c23316_g1_i1_(wsluc4)   460 VVVIGVPASDIDEDACACVQLVSGRKFDSASLRQYCKDYAQRDNENISGTK 510
81000_c2_seq1           460 VVVIGVPASDIDEDACACVQLVSGRKFDSASLRQYCKDYAQRDNENISGTK 510

81000_c2_seq2_(wsluc2)  511 TILPKHFLQFKEFPIIHAGKFDKLKIQQMAIERLNLGIFKKK          552
c23316_g1_i1_(wsluc4)   511 TIIPTYFLEFGEFPTIRGGKLDKLKMKQIAIERLDLED-RRK          551
81000_c2_seq1           511 TIIPTYFLEFGEFPTIRGGKLDKLKMKQIAIERLDLED-RRK          551
```

Supplementary Table S1. Summary statistics for sequencing

Sample	Raw reads	Trimmed, quality filtered reads	Trimmed, quality filtered reads (% of raw reads)
arm tip 1	53 244 178	39 715 694	74.6
arm tip 2	52 724 122	39 319 336	74.6
arm tip 3	53 663 020	39 991 398	74.5
arm tip 4	53 175 664	39 290 396	73.9
mantle 1	50 562 584	36 947 654	73.1
mantle 2	39 452 216	27 920 142	70.8
combined	302 821 784	223 184 620	73.7

Supplementary Table S2. *De novo* assembly statistics

Sample	Number of contigs	Number of bases assembled	N50 (bp)	Median contig length (bp)	Mean contig length (bp)	Maximum contig length (bp)
arm tip (four libraries merged)	178 970	148 602 829	1 728	371	830	21 209
mantle (two libraries merged)	53 406	36 531 652	1102	369	684	18 343
combined (all six libraries merged)	216 539	239 722 196	2 709	422	1 107	19 855

N50 = the length such that 50% of the assembled genome lies in N50 size or greater

Supplementary Table S7. Gene set enrichment analysis for most abundant arm tip tissue transcripts (p-value <0.05)

Gene ontology (GO) term	Number of transcripts listed under each GO term	Transcripts annotated with GO term	Adjusted p-value from the logistic regression
hydrogen ion transmembrane transport(GO:1902600)	7	comp21957_c0_seq1,comp33736_c0_seq2,comp58318_c0_seq1,comp70547_c1_seq1,comp72862_c1_seq1,comp86005_c0_seq1,comp86020_c0_seq1	1.29E-04
mitochondrial inner membrane(GO:0005743)	11	comp21962_c0_seq1,comp33755_c0_seq1,comp33766_c0_seq1,comp52493_c0_seq1,comp58318_c1_seq1,comp64797_c0_seq1,comp70028_c0_seq1,comp70547_c1_seq1,comp72862_c0_seq1,comp72862_c1_seq1,comp86006_c0_seq1	2.98E-04
cytochrome-c oxidase activity(GO:0004129)	5	comp33736_c0_seq2,comp58318_c0_seq1,comp72862_c1_seq1,comp86005_c0_seq1,comp86020_c0_seq1	3.70E-04
EC:1.9.3.1 (Cytochrome-c oxidase)	5	comp33736_c0_seq2,comp58318_c0_seq1,comp72862_c1_seq1,comp86005_c0_seq1,comp86020_c0_seq1	3.70E-04
respiratory chain(GO:0070469)	5	comp21955_c0_seq1,comp33766_c0_seq1,comp52526_c0_seq1,comp58318_c1_seq1,comp72862_c1_seq1	0.0010
iron ion binding(GO:0005506)	2	comp72862_c1_seq1,comp77255_c0_seq2	0.0013
integral component of membrane(GO:0016021)	16	comp21955_c0_seq1,comp22007_c0_seq1,comp33755_c0_seq1,comp33766_c0_seq1,comp52503_c0_seq1,comp52526_c0_seq1,comp52698_c1_seq1,comp58318_c0_seq1,comp58318_c1_seq1,comp64210_c0_seq1,comp70776_c0_seq1,comp72862_c1_seq1,comp75572_c0_seq1,comp76453_c0_seq1,comp79868_c4_seq1,comp86006_c0_seq1	0.0013
aerobic electron transport chain(GO:0019646)	1	comp58318_c0_seq1	0.0047
dense body(GO:0097433)	2	comp77978_c0_seq1,comp77978_c0_seq4	0.0051
mitochondrion(GO:0005739)	10	comp22005_c0_seq1,comp33612_c0_seq2,comp33739_c0_seq1,comp52454_c0_seq1,comp52536_c0_seq1,comp58318_c0_seq1,comp58323_c1_seq1,comp67934_c0_seq1,comp86011_c0_seq1,comp86020_c0_seq1	0.011
cytoskeleton(GO:0005856)	4	comp58323_c1_seq1,comp77978_c0_seq3,comp77978_c0_seq4,comp77978_c0_seq5	0.014
plasma membrane(GO:0005886)	12	comp21985_c0_seq1,comp33612_c0_seq2,comp33739_c0_seq1,comp33823_c0_seq1,comp52454_c0_seq1,comp52503_c0_seq1,comp52536_c0_seq1,comp61118_c0_seq1,comp72851_c0_seq1,comp77978_c0_seq1,comp77978_c0_seq4,comp79724_c6_seq4	0.017
respiratory chain complex III(GO:0045275)	1	comp70547_c1_seq1	0.025
ubiquinol-cytochrome-c reductase activity(GO:0008121)	1	comp70547_c1_seq1	0.025
electron carrier activity(GO:0009055)	1	comp70547_c1_seq1	0.025
EC:1.10.2.2 (Quinol--cytochrome-c reductase)	1	comp70547_c1_seq1	0.025
protease binding(GO:0002020)	2	comp33739_c0_seq1,comp52536_c0_seq1	0.026
mitochondrion transport along microtubule(GO:0047497)	2	comp33739_c0_seq1,comp52536_c0_seq1	0.026

membrane organization(GO:0061024)	2	comp33739_c0_seq1,comp52536_c0_seq1	0.026
regulation of proteasomal protein catabolic process(GO:0061136)	2	comp33739_c0_seq1,comp52536_c0_seq1	0.026
regulation of neuron death(GO:1901214)	2	comp33739_c0_seq1,comp52536_c0_seq1	0.026
positive regulation of intrinsic apoptotic signaling pathway by p53 class mediator(GO:1902255)	2	comp33739_c0_seq1,comp52536_c0_seq1	0.026
endosome membrane(GO:0010008)	3	comp33739_c0_seq1,comp33823_c0_seq1,comp52536_c0_seq1	0.031
endocytic vesicle membrane(GO:0030666)	3	comp33739_c0_seq1,comp33823_c0_seq1,comp52536_c0_seq1	0.031
mitotic G1 phase(GO:0000080)	3	comp33739_c0_seq1,comp33823_c0_seq1,comp52536_c0_seq1	0.031
G1/S transition of mitotic cell cycle(GO:0000082)	3	comp33739_c0_seq1,comp33823_c0_seq1,comp52536_c0_seq1	0.031
mitotic S phase(GO:0000084)	3	comp33739_c0_seq1,comp33823_c0_seq1,comp52536_c0_seq1	0.031
G2/M transition of mitotic cell cycle(GO:0000086)	3	comp33739_c0_seq1,comp33823_c0_seq1,comp52536_c0_seq1	0.031
mitotic anaphase(GO:0000090)	3	comp33739_c0_seq1,comp33823_c0_seq1,comp52536_c0_seq1	0.031
activation of MAPKK activity(GO:0000186)	3	comp33739_c0_seq1,comp33823_c0_seq1,comp52536_c0_seq1	0.031
protein polyubiquitination(GO:0000209)	3	comp33739_c0_seq1,comp33823_c0_seq1,comp52536_c0_seq1	0.031
stimulatory C-type lectin receptor signaling pathway(GO:0002223)	3	comp33739_c0_seq1,comp33823_c0_seq1,comp52536_c0_seq1	0.031
antigen processing and presentation of exogenous peptide antigen via MHC class I, TAP-dependent(GO:0002479)	3	comp33739_c0_seq1,comp33823_c0_seq1,comp52536_c0_seq1	0.031
MyD88-dependent toll-like receptor signaling pathway(GO:0002755)	3	comp33739_c0_seq1,comp33823_c0_seq1,comp52536_c0_seq1	0.031
glycogen biosynthetic process(GO:0005978)	3	comp33739_c0_seq1,comp33823_c0_seq1,comp52536_c0_seq1	0.031
transcription initiation from RNA polymerase II promoter(GO:0006367)	3	comp33739_c0_seq1,comp33823_c0_seq1,comp52536_c0_seq1	0.031
DNA damage response, signal transduction by p53 class mediator resulting in cell cycle arrest(GO:0006977)	3	comp33739_c0_seq1,comp33823_c0_seq1,comp52536_c0_seq1	0.031
epidermal growth factor receptor signaling pathway(GO:0007173)	3	comp33739_c0_seq1,comp33823_c0_seq1,comp52536_c0_seq1	0.031
transforming growth factor beta receptor signaling pathway(GO:0007179)	3	comp33739_c0_seq1,comp33823_c0_seq1,comp52536_c0_seq1	0.031
Notch signaling pathway(GO:0007219)	3	comp33739_c0_seq1,comp33823_c0_seq1,comp52536_c0_seq1	0.031
Notch receptor processing(GO:0007220)	3	comp33739_c0_seq1,comp33823_c0_seq1,comp52536_c0_seq1	0.031
I-kappaB kinase/NF-kappaB signaling(GO:0007249)	3	comp33739_c0_seq1,comp33823_c0_seq1,comp52536_c0_seq1	0.031
JNK cascade(GO:0007254)	3	comp33739_c0_seq1,comp33823_c0_seq1,comp52536_c0_seq1	0.031
Ras protein signal transduction(GO:0007265)	3	comp33739_c0_seq1,comp33823_c0_seq1,comp52536_c0_seq1	0.031
circadian rhythm(GO:0007623)	3	comp33739_c0_seq1,comp33823_c0_seq1,comp52536_c0_seq1	0.031
insulin receptor signaling pathway(GO:0008286)	3	comp33739_c0_seq1,comp33823_c0_seq1,comp52536_c0_seq1	0.031

regulation of necrotic cell death(GO:0010939)	3	comp33739_c0_seq1,comp33823_c0_seq1,comp52536_c0_seq1	0.031
endosomal transport(GO:0016197)	3	comp33739_c0_seq1,comp33823_c0_seq1,comp52536_c0_seq1	0.031
virion assembly(GO:0019068)	3	comp33739_c0_seq1,comp33823_c0_seq1,comp52536_c0_seq1	0.031
viral protein processing(GO:0019082)	3	comp33739_c0_seq1,comp33823_c0_seq1,comp52536_c0_seq1	0.031
negative regulation of transforming growth factor beta receptor signaling pathway(GO:0030512)	3	comp33739_c0_seq1,comp33823_c0_seq1,comp52536_c0_seq1	0.031
anaphase-promoting complex-dependent proteasomal ubiquitin-dependent protein catabolic process(GO:0031145)	3	comp33739_c0_seq1,comp33823_c0_seq1,comp52536_c0_seq1	0.031
negative regulation of type I interferon production(GO:0032480)	3	comp33739_c0_seq1,comp33823_c0_seq1,comp52536_c0_seq1	0.031
positive regulation of type I interferon production(GO:0032481)	3	comp33739_c0_seq1,comp33823_c0_seq1,comp52536_c0_seq1	0.031
tumor necrosis factor-mediated signaling pathway(GO:0033209)	3	comp33739_c0_seq1,comp33823_c0_seq1,comp52536_c0_seq1	0.031
toll-like receptor 2 signaling pathway(GO:0034134)	3	comp33739_c0_seq1,comp33823_c0_seq1,comp52536_c0_seq1	0.031
toll-like receptor 3 signaling pathway(GO:0034138)	3	comp33739_c0_seq1,comp33823_c0_seq1,comp52536_c0_seq1	0.031
toll-like receptor 4 signaling pathway(GO:0034142)	3	comp33739_c0_seq1,comp33823_c0_seq1,comp52536_c0_seq1	0.031
toll-like receptor 5 signaling pathway(GO:0034146)	3	comp33739_c0_seq1,comp33823_c0_seq1,comp52536_c0_seq1	0.031
toll-like receptor 9 signaling pathway(GO:0034162)	3	comp33739_c0_seq1,comp33823_c0_seq1,comp52536_c0_seq1	0.031
toll-like receptor 10 signaling pathway(GO:0034166)	3	comp33739_c0_seq1,comp33823_c0_seq1,comp52536_c0_seq1	0.031
ion transmembrane transport(GO:0034220)	3	comp33739_c0_seq1,comp33823_c0_seq1,comp52536_c0_seq1	0.031
TRIF-dependent toll-like receptor signaling pathway(GO:0035666)	3	comp33739_c0_seq1,comp33823_c0_seq1,comp52536_c0_seq1	0.031
Fc-epsilon receptor signaling pathway(GO:0038095)	3	comp33739_c0_seq1,comp33823_c0_seq1,comp52536_c0_seq1	0.031
toll-like receptor TLR1:TLR2 signaling pathway(GO:0038123)	3	comp33739_c0_seq1,comp33823_c0_seq1,comp52536_c0_seq1	0.031
toll-like receptor TLR6:TLR2 signaling pathway(GO:0038124)	3	comp33739_c0_seq1,comp33823_c0_seq1,comp52536_c0_seq1	0.031
negative regulation of epidermal growth factor receptor signaling pathway(GO:0042059)	3	comp33739_c0_seq1,comp33823_c0_seq1,comp52536_c0_seq1	0.031
error-prone translesion synthesis(GO:0042276)	3	comp33739_c0_seq1,comp33823_c0_seq1,comp52536_c0_seq1	0.031
DNA damage response, detection of DNA damage(GO:0042769)	3	comp33739_c0_seq1,comp33823_c0_seq1,comp52536_c0_seq1	0.031
positive regulation of I-kappaB kinase/NF-kappaB signaling(GO:0043123)	3	comp33739_c0_seq1,comp33823_c0_seq1,comp52536_c0_seq1	0.031
vascular endothelial growth factor receptor signaling pathway(GO:0048010)	3	comp33739_c0_seq1,comp33823_c0_seq1,comp52536_c0_seq1	0.031
neurotrophin TRK receptor signaling pathway(GO:0048011)	3	comp33739_c0_seq1,comp33823_c0_seq1,comp52536_c0_seq1	0.031
T cell receptor signaling pathway(GO:0050852)	3	comp33739_c0_seq1,comp33823_c0_seq1,comp52536_c0_seq1	0.031

negative regulation of ubiquitin-protein ligase activity involved in mitotic cell cycle(GO:0051436)	3	comp33739_c0_seq1,comp33823_c0_seq1,comp52536_c0_seq1	0.031
positive regulation of ubiquitin-protein ligase activity involved in regulation of mitotic cell cycle transition(GO:0051437)	3	comp33739_c0_seq1,comp33823_c0_seq1,comp52536_c0_seq1	0.031
regulation of transcription from RNA polymerase II promoter in response to hypoxia(GO:0061418)	3	comp33739_c0_seq1,comp33823_c0_seq1,comp52536_c0_seq1	0.031
necroptotic process(GO:0070266)	3	comp33739_c0_seq1,comp33823_c0_seq1,comp52536_c0_seq1	0.031
nucleotide-binding oligomerization domain containing signaling pathway(GO:0070423)	3	comp33739_c0_seq1,comp33823_c0_seq1,comp52536_c0_seq1	0.031
error-free translesion synthesis(GO:0070987)	3	comp33739_c0_seq1,comp33823_c0_seq1,comp52536_c0_seq1	0.031
intracellular transport of virus(GO:0075733)	3	comp33739_c0_seq1,comp33823_c0_seq1,comp52536_c0_seq1	0.031
negative regulation of canonical Wnt signaling pathway(GO:0090090)	3	comp33739_c0_seq1,comp33823_c0_seq1,comp52536_c0_seq1	0.031
positive regulation of canonical Wnt signaling pathway(GO:0090263)	3	comp33739_c0_seq1,comp33823_c0_seq1,comp52536_c0_seq1	0.031
hydrogen ion transmembrane transporter activity(GO:0015078)	3	comp52493_c0_seq1,comp72862_c0_seq1,comp79868_c4_seq1	0.034
positive regulation of NF-kappaB transcription factor activity(GO:0051092)	4	comp33739_c0_seq1,comp33823_c0_seq1,comp52536_c0_seq1,comp70028_c0_seq1	0.039
negative regulation of transcription from RNA polymerase II promoter(GO:0000122)	4	comp33739_c0_seq1,comp33823_c0_seq1,comp52536_c0_seq1,comp64529_c0_seq1	0.045
positive regulation of transcription from RNA polymerase II promoter(GO:0045944)	4	comp33739_c0_seq1,comp33823_c0_seq1,comp52536_c0_seq1,comp64529_c0_seq1	0.045

Supplementary Table S8. Gene set enrichment analysis for most abundant mantle tissue transcripts (p-value <0.05)

Gene ontology (GO) term	Number of transcripts listed under each GO term	Transcripts annotated with GO term	Adjusted p-value from the logistic regression
hydrogen ion transmembrane transport(GO:1902600)	11	comp21938_c0_seq1,comp21956_c0_seq1,comp21957_c0_seq1,comp33736_c0_seq1,comp33736_c0_seq2,comp58318_c0_seq1,comp70547_c1_seq1,comp72862_c1_seq1,comp78413_c0_seq1,comp86005_c0_seq1,comp86020_c0_seq1	6.03E-06
apoptotic process(GO:0006915)	4	comp67767_c0_seq1,comp70028_c0_seq1,comp77978_c0_seq1,comp77978_c0_seq2	9.83E-05
membrane raft(GO:0045121)	4	comp61118_c0_seq1,comp71176_c0_seq1,comp77978_c0_seq1,comp77978_c0_seq2	1.15E-04
ATP binding(GO:0005524)	18	comp21962_c0_seq1,comp21972_c0_seq1,comp34825_c0_seq1,comp35191_c0_seq1,comp58316_c1_seq1,comp58316_c1_seq2,comp58338_c0_seq1,comp61118_c0_seq1,comp70776_c0_seq1,comp71176_c0_seq1,comp73342_c0_seq1,comp76442_c0_seq4,comp77978_c0_seq1,comp77978_c0_seq2,comp77978_c0_seq5,comp80128_c1_seq4,comp83466_c0_seq1,comp86007_c0_seq1	1.22E-04
nematode larval development(GO:0002119)	3	comp67767_c0_seq1,comp77978_c0_seq1,comp77978_c0_seq2	1.78E-04
embryo development ending in birth or egg hatching(GO:0009792)	3	comp67767_c0_seq1,comp77978_c0_seq1,comp77978_c0_seq2	1.78E-04
growth(GO:0040007)	3	comp67767_c0_seq1,comp77978_c0_seq1,comp77978_c0_seq2	1.78E-04
hermaphrodite genitalia development(GO:0040035)	3	comp67767_c0_seq1,comp77978_c0_seq1,comp77978_c0_seq2	1.78E-04
cytochrome-c oxidase activity(GO:0004129)	8	comp21938_c0_seq1,comp33736_c0_seq1,comp33736_c0_seq2,comp58318_c0_seq1,comp72862_c1_seq1,comp78413_c0_seq1,comp86005_c0_seq1,comp86020_c0_seq1	1.78E-04
EC:1.9.3.1 (Cytochrome-c oxidase)	8	comp21938_c0_seq1,comp33736_c0_seq1,comp33736_c0_seq2,comp58318_c0_seq1,comp72862_c1_seq1,comp78413_c0_seq1,comp86005_c0_seq1,comp86020_c0_seq1	1.78E-04
ATP synthesis coupled proton transport(GO:0015986)	7	comp21951_c0_seq1,comp21962_c0_seq1,comp72862_c0_seq1,comp86007_c0_seq1,comp86016_c0_seq1,comp86017_c0_seq1,comp86040_c0_seq1	3.02E-04
mitochondrial inner membrane(GO:0005743)	23	comp21953_c0_seq1,comp21956_c0_seq1,comp21962_c0_seq1,comp21993_c0_seq1,comp33755_c0_seq1,comp33766_c0_seq1,comp52116_c0_seq1,comp52493_c0_seq1,comp58086_c0_seq2,comp58318_c1_seq1,comp64797_c0_seq1,comp69790_c0_seq1,comp70028_c0_seq1,comp70547_c1_seq1,comp71205_c1_seq1,comp72862_c0_seq1,comp72862_c1_seq1,comp72977_c0_seq9,comp75866_c1_seq1,comp81964_c1_seq1,comp86006_c0_seq1,comp86032_c0_seq1,comp86040_c0_seq1	3.34E-04
structural constituent of cytoskeleton(GO:0005200)	7	comp52168_c0_seq1,comp72811_c1_seq1,comp72811_c1_seq3,comp75065_c0_seq1,comp76300_c0_seq3,comp77978_c0_seq1,comp77978_c0_seq2	3.98E-04
integral component of membrane(GO:0016021)	22	comp21955_c0_seq1,comp21972_c0_seq1,comp22007_c0_seq1,comp33316_c0_seq1,comp33755_c0_seq1,comp33766_c0_seq1,comp35191_c0_seq1,comp52503_c0_seq1,comp52526_c0_seq1,comp52622_c0_seq1,comp58318_c0_seq1,comp58318_c1_seq1,comp58363_c0_seq1,comp70776_c0_seq1,comp72768_c0_seq3,comp72768_c0_seq4,comp72768_c0_seq7,comp72862_c1_seq1,comp78756_c1_seq2,comp86006_c0_seq1,comp86042_c0_seq1,comp86048_c0_seq1	0.0034
aerobic respiration(GO:0009060)	2	comp52450_c0_seq2,comp72862_c1_seq1	0.0044
heme binding(GO:0020037)	3	comp58230_c0_seq1,comp72862_c1_seq1,comp84838_c1_seq1	0.0070
aerobic electron transport chain(GO:0019646)	1	comp58318_c0_seq1	0.0082
dense body(GO:0097433)	1	comp77978_c0_seq1	0.0090
iron ion binding(GO:0005506)	1	comp72862_c1_seq1	0.0091

oxidative phosphorylation(GO:0006119)	1	comp72862_c1_seq1	0.0091
cytoplasmic membrane-bounded vesicle(GO:0016023)	11	comp21962_c0_seq1,comp21985_c0_seq1,comp52535_c0_seq1,comp58314_c0_seq1,comp58353_c0_seq1,comp61054_c0_seq2,comp68003_c0_seq1,comp68003_c0_seq2,comp71278_c0_seq1,comp71278_c0_seq2,comp81019_c4_seq1	0.0097
respiratory chain(GO:0070469)	8	comp21955_c0_seq1,comp21956_c0_seq1,comp33766_c0_seq1,comp52526_c0_seq1,comp58230_c0_seq1,comp58318_c1_seq1,comp71205_c1_seq1,comp72862_c1_seq1	0.011
proton-transporting two-sector ATPase complex, proton-transporting domain(GO:0033177)	1	comp21957_c0_seq1	0.013
proton-transporting ATP synthase activity, rotational mechanism(GO:0046933)	4	comp21962_c0_seq1,comp86007_c0_seq1,comp86016_c0_seq1,comp86017_c0_seq1	0.014
ATP hydrolysis coupled proton transport(GO:0015991)	2	comp21962_c0_seq1,comp86007_c0_seq1	0.015
proton-transporting ATP synthase complex, catalytic core F(1)(GO:0045261)	3	comp21962_c0_seq1,comp86007_c0_seq1,comp86017_c0_seq1	0.016
hydrogen ion transmembrane transporter activity(GO:0015078)	3	comp52493_c0_seq1,comp72862_c0_seq1,comp86040_c0_seq1	0.016
respiratory chain complex III(GO:0045275)	1	comp70547_c1_seq1	0.018
striated muscle thin filament(GO:0005865)	2	comp77978_c0_seq1,comp77978_c0_seq2	0.022
actin filament(GO:0005884)	2	comp77978_c0_seq1,comp77978_c0_seq2	0.022
cell cortex(GO:0005938)	2	comp77978_c0_seq1,comp77978_c0_seq2	0.022
ATPase activity(GO:0016887)	2	comp77978_c0_seq1,comp77978_c0_seq2	0.022
mitotic cytokinesis(GO:0000281)	2	comp77978_c0_seq1,comp77978_c0_seq2	0.022
receptor-mediated endocytosis(GO:0006898)	2	comp77978_c0_seq1,comp77978_c0_seq2	0.022
meiotic nuclear division(GO:0007126)	2	comp77978_c0_seq1,comp77978_c0_seq2	0.022
inductive cell migration(GO:0040039)	2	comp77978_c0_seq1,comp77978_c0_seq2	0.022
striated muscle myosin thick filament assembly(GO:0071688)	2	comp77978_c0_seq1,comp77978_c0_seq2	0.022
EC:3.6.1.3 (Adenosinetriphosphatase)	2	comp77978_c0_seq1,comp77978_c0_seq2	0.022
arginine kinase activity(GO:0004054)	2	comp58316_c1_seq1,comp58316_c1_seq2	0.023
EC:2.7.3.3 (Arginine kinase)	2	comp58316_c1_seq1,comp58316_c1_seq2	0.023
myosin complex(GO:0016459)	4	comp52535_c0_seq1,comp81019_c4_seq1,comp81712_c3_seq1,comp81712_c3_seq2	0.030
calcium ion binding(GO:0005509)	4	comp52535_c0_seq1,comp73847_c0_seq1,comp79899_c1_seq1,comp81019_c4_seq1	0.031
ubiquinol-cytochrome-c reductase activity(GO:0008121)	2	comp21956_c0_seq1,comp70547_c1_seq1	0.039
EC:1.10.2.2 (Quinol--cytochrome-c reductase)	2	comp21956_c0_seq1,comp70547_c1_seq1	0.039
